# The Role of the Ultrasonographic Knee Assessment in the Diagnosis of Patellofemoral Pain Syndrome: Ultrasonography and Patellofemoral Pain Syndrome

**DOI:** 10.31661/gmj.v11i.2420

**Published:** 2022-12-25

**Authors:** Shila Haghighat, Mehdi Karami, Nasim Ghasemi, Babak Vahdatpour, Samira Soleimany

**Affiliations:** ^1^ Department of Physical Medicine and Rehabilitation, School of Medicine, Isfahan University of Medical Sciences, Isfahan, Iran; ^2^ Department of Imaging and Radiology, Isfahan University of Medical Sciences, Isfahan, Iran

**Keywords:** Patellofemoral Pain Syndrome, Sulcus, Q Angle, Ultrasonography

## Abstract

**Background:**

Patellofemoral Pain Syndrome (PFPS) is a common anterior knee compartment pain etiology. It has been aimed to assess the ultrasonographic findings of the patellofemoral joint in patients with PFPS versus healthy individuals.

**Materials and Methods:**

The current case-control investigation was performed on 30 cases suffering from patellofemoral joint pain and 30 healthy individuals in Isfahan during 2020-21. All cases underwent ultrasonography to assess cartilage thickness, sulcus angle, and sulcus depth. We also measured the Q angle with a manual goniometer.

**Results:**

In healthy individuals, Q angle scores were statistically lower than in the cases group (P=0.002). The sulcus angle was remarkably higher among the patients compared to the controls. The cartilage thickness (P=0.88) and sulcus depth (P=0.543) scores had no statistical difference between the PFPS and healthy subjects (P0.05).

**Conclusion:**

Patients with PFPS had significantly higher Q angle and lower sulcus angle than the healthy controls.

## Introduction

Patellofemoral Pain Syndrome (PFPS) is a common cause of anterior knee compartment pain [1][2]. PFPS is diagnosed according to the pain complaint in the anterior part of the knee involving the patella, retinaculum, and surrounding soft tissues; however, a definite diagnosis should be made by excluding the other pathologies inside and outside the knee [3]. This syndrome mainly occurs due to excessive and inappropriate use of the knee joints, whereas direct trauma to the knee is the underlying etiology in limited cases [4]. PFPS involves 22000 cases per year and predominantly involves women with a two-fold increased risk [5]. Surfing the literature has demonstrated that PFPS is responsible for over 25% of knee pain in young adults and more than 75% of all causes of anterior knee pain [6][7].

The exact etiology of pain in PFPS remained unclear; however, the existing hypothesis claims that pain originates from the junction of extensor muscles, retinacula, Hoffa fat, and subchondral bone [1][8]. Disorders in the knee extensor mechanism are the other theory regarding the pathophysiology of PFPS. Accordingly, individuals with inappropriate knee extensor mechanisms are at increased risk for recurrent patella dislocation or persistent anterior knee pain [3][9]. The extensor mechanism refers to components including the extensor muscles of the knee, quadriceps tendon, patellofemoral joint, patellar tendon, and tibial tubercle [10]. Any chronic pathology in this area leads to adversities ranging from tendinopathy and joint surface lesions to patellofemoral maltracking [11].

The clinical manifestation of PFPS includes pain in the forward knee movement in a young person [12]. The pain is often bilateral and worsens with movements such as climbing stairs and squatting. In PFPS, the patella is usually at rest, or the patella moves in the opposite position when the knee is moving [3].

Imaging modalities have been applied to diagnose knee pathologies. These modalities are divided into two categories of static and dynamic images. Static images such as radiographs and computed tomography scans cannot examine the patella's movements and location during muscle contractions. In contrast, ultrasound studies as dynamic ones can measure muscle and bone's kinematic properties and relationships during movements [13]. Ultrasound can also assess the soft tissues surrounding the patella, the trochlear groove, and its connection to the patella tip [14].

Magnetic resonance imaging (MRI) and ultrasonography are the primary imaging modalities applied to study knee extensor mechanisms. Given that, ultrasonography has provided numerous quantitative and qualitative criteria for PFPS. Although MRI is the best modality for examining patellar dislocation, nearby soft tissue and tendons, in particular, are appropriately visualized in ultrasound assessments [15][16]; however, the knowledge and practice regarding ultrasonography use for PFPS are limited.

A study by Fanlo-Mazas et al. demonstrated that ultrasonography study of the knees provided valuable information; therefore, ultrasound could have high clinical values in this regard [17]. Other studies highlighted the use of ultrasound images in diagnosing and following up the patients with knee pain and emphasized the convenience, cost-effectiveness, and value of this modality for clinical practice [18][19].

Due to the simplicity, low cost, and reproducibility of ultrasound and lacking adequate information regarding the use of ultrasonography in PFPS, the current study aims to assess ultrasonographic characteristics of the patellofemoral joint in patients with PFPS.

## Materials and Methods

### Study Population

The current case-control study has been conducted on 30 patients with anterior knee compartment pain and 30 age- and gender-matched subjects with complaints other than knee pain referring to the Isfahan University of Medical Sciences affiliated outpatient clinics of physical medicine and rehabilitation from May 2020 to June 2021.

### Sample Size Calculation

The study population was calculated according to the following formula in which Z_1_=1.96 (95% confidence interval), Z_2_=0.84 (80% power coefficient), S_1_=4.4, S_2_=3.5, m_1_=17.4, and m_2_=14.6. According to the formula mentioned, the total number of patients equaled 60 in two 30-participant groups.


n = \frac{(Z_1 + Z_2)^2(S_1^2 + S_2^2)}{(m_1 - m_2)^2}

### Inclusion and Exclusion Criteria

Patients aged 15 to 45 years with patellofemoral joint pain lasting for the least three months and pain severity deterioration with activity, including knee flexion were included. Signs and symptoms of knee joint instability, anatomical abnormalities such as osteoarthritis, signs of ligament or tendon injury, degenerative pathologies of the patellofemoral joint, history of trauma to the knee, pelvis, or lumbar spine in the previous three months, and a history of metabolic bone diseases were determined as the exclusion criteria.

### Data Collection

The subjects' demographic characteristics, including age, gender, and anthropometric indices (height and weight), were entered into the study checklist. Body mass index (BMI) was calculated by the division of weight (kg) by the height square (meter). Besides, the cases' unilateral versus bilateral anterior knee pain complaints were recorded.

Ultrasonography examination was performed for all the subjects, and variables, including the sulcus angle, sulcus depth, and cartilage thickness were assessed. Also, the Q angle was measured by a manual goniometer. All ultrasound measurements were performed using a Supersonix ultrasound device (Aixplorer™, Supersonic Imagine, Aix-en-Provence, France) with a 14-17 MHz linear transducer. A target expert radiologist in musculoskeletal ultrasound performed all the ultrasonographic studies to minimize the potential bias.

There are two transverse and vertical approaches for measuring the thickness of the cartilage. Herein, the transverse approach has been applied. Accordingly, after resting for 10 minutes, the patient was placed on a chair, and the knee was placed in a 90-degree flexion position. The transducer was placed transversely on the middle part of the inner condyle of the femur, in which three lines are drawn from the layer on the surface of the soft tissue to the bone. The average distance between these three lines was considered as the thickness of the knee cartilage. In the vertical approach, the patient was placed on a chair, and the desired knee was in full flexion (133 to 151 degrees). The transducer was placed vertically on the line between the patella's inner edge and the femur's inner condyle.

The patient was placed in the upraise position to assess the Q angle using a goniometer. The landmarks, including the anterior superior iliac spine (ASIS), the mid-parts of the patella, and the tibia, were marked. Q angle was measured from the top by placing one goniometer arm on the ASIS, the midpoint of the goniometer on the midpoint of the patella, and the other arm on the tibia. The acute angle between the two arms was the Q angle.

### Ethical Consideration

The study protocol that met the tenets of the Helsinki declaration and was approved by ethics committee of Isfahan University of Medical Sciences (code number: IR.MUI.MED.REC.1398.371). The study was explained to the participants, and they were reassured regarding their information confidentiality and signed written consent.

### Statistical Analysis

The gathered data was entered into the IBM SPSS Statistics for Windows, version 23 (IBM Corp., Armonk, NY., USA). The mean±standard deviation was applied for the primary assessment of the continuous data, and the nominal variables were presented as absolute numbers and percentages. The independent t-test and Chi-square test were applied to analyze the quantitative and qualitative variables. A P-value=0.05 was considered the level of significance.

## Results

**Table:** Table1. Demographic Characteristics of Patients with PFPS and Healthy Individuals

**Variables**	**Groups**		**P-value**
	**Case (n=30)**	**Control (n=30)**	
**Age, y (mean±SD)**	32.77±9.11	31.67±8.11	0.623
**BMI, Kg/m^2^(mean±SD) **	27.82±3.91	26.14±3.58	0.087
**Gender, n(%)**			
Female	20 (66.7)	13 (43.3)	0.069
Male	10 (33.3)	17 (56.7)

**Table:** Table2. Ultrasound Findings of Patellofemoral Joint in Patients with PFPS and Healthy Individuals

**Variables**	**Groups**		**P-value**
	**Case (n=30)**	**Control (n=30)**	
**Cartilage thickness, (mean±SD) **	2.65±0.73	2.61±0.51	0.808
**Sulcus angle, (mean±SD) **	140.90±9.5	146.13±3.52	0.006
**Sulcus depth, (mean±SD) **	4.42±1.38	4.42±0.82	0.543

The current case-control investigation has been conducted on 60 individuals undergoing ultrasonography of the knee. The mean age of the studied population was 30.29±6.51 years (range: 15-43). The patients suffering from PFPs had a mean age of 32.77±9.11 years (range: 15-43) and a mean BMI of 27.82±3.91 kg/m2 (range: 21-35). As the showed in Table-1, most patients in the case group were female (66.7%), and the healthy group was male (56.7%). Also, there were no significant differences in the term of age, BMI, and gender between groups (Table-1).

Ultrasound findings in the healthy subjects versus the patients are demonstrated in Table-2. The independent t-test revealed that the sulcus angle was statistically different between the two groups (P=0.006). However, cartilage thickness (P=0.88) and sulcus depth (P=0.543) showed no remarkable difference between the case and control groups. The mean Q angle in case and control groups was 19.23±1.46 and 17.93±1.7, respectively. Also, significant higher amounts of Q angle were observed in the case group (P=0.002).

## Discussion

In the current study, we found significantly higher Q angle and lower sulcus angle among the patients with PFPS compared to the healthy subjects. However, the other parameters, including the trochlear depth, trochlear angle, and the position of the patella, were not different between the cases and controls. Disorders in these parameters could easily lead to knee instability and PFPS.

Using ultrasound indices for musculoskeletal syndromes, including PFPS, could have clinical importance because this imaging modality is easy to access, harmless and cost-effective. As a result, constant efforts have been made in this regard. In 2008, a survey was performed by Brushøj et al. on 30 army recruits with patellofemoral pain in Denmark [20]. Ultrasound and MRI assessments revealed that pain expanded in all synovial surfaces was the main sign of this syndrome, and changes in the sulcus angle were remarkable among the patients suffering from PFPS [20]. Another study by Lin et al. [21] evaluated ultrasonography imaging of 89 individuals with PFPS. Their findings were consistent with ours as they demonstrated that the Q angle was negatively correlated with the vastus medialis obliquus insertion ratio and the volume of this muscle [21]. They also mentioned that the sulcus angle of the patella was significantly reduced in individuals suffering from PFPS [21].

Payne et al. evaluated the association of gluteus medius muscle thickness with the ultrasound findings of patients with patellofemoral pain. In contrast to our findings, they represented that the thickness of the gluteus medius muscle both at rest or when contracted, and the gluteus medius muscle activation and Q angle were not remarkably different in the subjects with PFPS compared to the healthy subjects. However, the sulcus angle was statistically less in the patients with knee pain, which agrees with our study [22].

Another survey was performed in the Netherlands in which the ultrasound imaging of patients with unilateral PFPS versus healthy controls was assessed [23]. This study showed that the sulcus angle and Q angle, along with other characteristics such as the thickness of the lateral retinaculum, could play an essential role in diagnosing, treating, and following up on patients with anterior knee pain [23]. Furthermore, they insisted on the significance of the lateral retinaculum and patellar cartilage in the pathophysiology of PFPS [23].

Fischhoff et al. showed that the cartilaginous trochlear angle, patellar tip to trochlear groove distance, and dysplasia in this region might be critical factors in ultrasound studies of patients with PFPS [24]. However, the parameters assessed, including Q angle, sulcus angle and depth, and thickness of the lateral retinaculum, require further investigation [24].

Although we showed that the cartilage thickness and sulcus depth were not different between the cases and controls, the Q and sulcus angles assessments could have clinical significance for this condition. The small sample size and not evaluating other indices, including the thickness of the gluteus medius muscle, its activation, or the thickness of the lateral retinaculum, were our study's limitations. The lack of assessing >the lateral subluxation of the patella due to the lack of ultrasound equipment for this means was the other limitation of the current study. Hence, further multi-centric studies on larger populations are recommended.

## Conclusion

Patients with PFPS had significantly higher Q angles and lower sulcus angles than the healthy controls. Hence the routine use of ultrasonography to assess knee pains is strongly recommended.

## Conflict of Interest

The authors declare that they have no competing interests.
